# Fermented total mixed ration enhances nutrient digestibility and modulates the milk components and fecal microbial community in lactating Holstein dairy cows

**DOI:** 10.3389/fvets.2024.1408348

**Published:** 2024-08-14

**Authors:** Lijun Wang, Sanjun Jin, Ping Wang, Xinxin Li, Chaoqi Liu, Siying Sun, Guangning Zhang, Juan Chang, Qingqiang Yin, Haiyang Zhang, Qun Zhu

**Affiliations:** ^1^College of Animal Science and Technology, Henan Agricultural University, Zhengzhou, China; ^2^Institute of International Education, Henan Agricultural University, Zhengzhou, China; ^3^College of Animal Science and Technology, Northeast Agricultural University, Harbin, China; ^4^Henan Delin Biological Products Co., Ltd., Xinxiang, China

**Keywords:** fermented total mixed ration, dairy cows, digestibility, fecal microbiota, fecal fermentation pattern

## Abstract

Fermented total mixed ration (FTMR) is an effective method of preserving high-moisture byproducts with higher aerobic stability after fermentation. FTMR has the potential to fulfill the daily nutritional requirements of cattle and enhance their production performance. The objective of this research was to examine the influence of FTMR on lactation performance, total tract apparent digestibility, fecal microbiota communities, and fermentation profiles in lactating dairy cows. A total of 12 cows were randomly assigned into two groups: the TMR group and the FTMR group. The TMR group was fed a total mixed ration (TMR) diet, and the FTMR group was fed an FTMR diet. The FTMR did not impact milk yield in dairy cows despite a decrease in dry matter intake, which increased the efficiency of the feed. In contrast to that in the TMR group, the milk fat content in the FTMR group was greater. The FTMR group showed greater digestibility of neutral detergent fiber (NDF), organic matter (OM), dry matter (DM), crude protein (CP), and acid detergent fiber (ADF) in the total digestive tract than did the TMR group. The FTMR increased the concentration of butyrate in the fecal matter and reduced the pH of the feces. The Chao1, ACE, and Shannon indices of the archaeal community in dairy cow feces were significantly higher in cow fed the FTMR compared to those fed the TMR. LefSe analysis revealed higher levels of *Oscillospira*, *Lactobacillus*, *Prevotella*, and *Dehalobacterium* in the feces of dairy cows fed the FTMR than in those fed the TMR. However, the abundances of *Roseburia*, *rc4-4*, *Bulleidia* and *Sharpea* exhibited the opposite trend. The abundances of *Halobacteria*, *Halobacteriales*, and *Halobacteriaceae*, which are biomarkers for distinguishing fecal archaea in the TMR from the FTMR, were substantially greater in the feces of dairy cows that consumed the TMR than in those that consumed the FTMR. Therefore, FTMR can improve the milk fat content, total tract apparent feed digestibility efficiency, and diversity of archaea in the feces. Additionally, this work provides a theoretical basis for the feasibility of FTMR feeding for dairy cows.

## 1 Introduction

One of the primary challenges confronting the cattle industry is the deficiency of high-quality protein and roughage resources in China (1). Despite the presence of abundant unconventional feed resources and crop residues in China, their digestion and utilization rates as feed resources remain low (2). Fermented total mixed ration (FTMR) technology represents a novel feeding approach developed from TMR modulation and processing technology (3). It effectively utilizes unconventional feed resources and crop residues as feed sources, minimizing waste, which helps mitigate feed resource shortages and reduce breeding costs (4). A high-quality diet with comprehensive, balanced nutrition and long-term storage is achieved by thoroughly mixing and fermenting various feed materials, including forage, concentrate, and essential minerals, with fermentation bacteria (5). After fermentation, the feed demonstrates high levels of crude protein, excellent palatability, and significant nutritive value (6, 7). FTMR can effectively utilize unconventional feed resources and crop residues as feed sources to reduce waste, thereby alleviating feed resource shortages and lowering breeding costs.

Due to the intricate conditions of anaerobic fermentation in FTMR, their microbial composition is highly diverse. Therefore, the microbial makeup of feed plays a critical role not only in fermentation quality but also in the aerobic stability of the feed (8). With advancements in microbial technology applied in the feed industry, the use of enzymes and bacteria has become a common strategy to enhance silage feed quality (9). Optimal combinations of enzymes and bacteria can effectively boost the fermentation of feed materials, improving both the quality and feeding efficiency (10–12). Cellulase breaks down cellulose, hemicellulose, and lignin in feed into accessible sugars that lactic acid bacteria can utilize, increasing their fermentation substrate, accelerating pH reduction, and enhancing silage fermentation quality (13, 14). *Lactobacillus buchneri*, a heterotypic fermentation bacterium, not only enhances aerobic stability and prolongs shelf life but also improves animal gut health and immunity, and increases milk production in cows (15).

In ruminants, the gastrointestinal microbes of dairy cows are pivotal for acquiring nutrients and energy (16, 17). Research has shown that diet largely determines the composition of bacteria in feces and the structure of the archaeal community in dairy cows, with the fecal microbiome serving as an indicator of hindgut microbial changes (18–20). Diets high in concentrates significantly decrease the populations of cellulolytic bacteria such as *Ruminococcus*, *Fibrobacter*, and *Ruminiclostridium*, as well as methanogens like *Methanosarcina*, *Methanobrevibacter*, and *Methanosphaera* (21). Al-Azzawi et al. demonstrated that adding powdered activated carbon to the diet reduces the abundance of *Proteobacteria* and *Methanobrevibacter* in feces (21). Additionally, hindgut archaea are responsible for methane production, which contributes significantly to global warming (22–24). However, limited information exists on the effects of co-fermentation TMR with *Lactobacillus brucei* and cellulase on fecal fermentation parameters and microbial diversity. This study aimed to evaluate and compare the impacts of total mixed rations (TMRs) and fermented total mixed rations (FTMR) on lactation performance, fecal fermentation parameters, and fecal microbiota in dairy cattle, providing valuable insights into the use of FTMR for dairy cow management.

## 2 Materials and methods

### 2.1 Animals and diets

All animal experimental procedures were approved by the Animal Care and Use Committee of Henan Agricultural University (Approval number: HENAU-2021-025).

Experimental dairy cows were obtained from Ruiya Dairy Farm located in Zhengzhou, Henan, China. A total of twelve parity 2 Holstein cows (average body weight = 616 ± 13.4 kg, average lactation period = 106 ± 7.55 days) were randomly assigned to either the total mixed ration (TMR, *n* = 6) or fermented total mixed ration (FTMR, *n* = 6) groups based on their daily milk yield and lactation period. Each group included 6 replicates (pens) with one cow per pen. The study was conducted over a period of 14 weeks, with the initial two weeks designated as an adaptation period.

Both the TMR and FTMR diets consisted of 50% forage and 50% concentrate on a dry matter basis, formulated to meet nutritional requirements using the Cornell-Penn-Miner Dairy model version 3.08.01. The compositions of TMR and FTMR were identical (on a dry matter basis): alfalfa hay (11.80%), wet corn gluten feed (6.86%), corn stover (3.43%), corn silage (27.80%), ground corn (24.60%), soybean meal (9.80%), cottonseed meal (4.91%), DDGS (7.35%), expanded soybean (0.95%), and premix (2.50%). The moisture content was adjusted to 48.0%. Lactic acid bacteria (LAB) and cellulase were added to the FTMR. LAB included a blend of *Lactobacillus plantarum* (CGMCC 1.12934, obtained from the China General Microbiological Culture Collection Center) and *Lactobacillus brucei* (BNCC189797, obtained from Beina Bio, Beijing, China) in a 1:1 ratio, applied at 1 × 10^11^ cfu/g of fresh material. Cellulase (10,000 U/g, XS Biotechnology Co., Ltd., Beijing, China) was added at 10 g/kg of fresh material. Additives were dissolved in water and uniformly sprayed onto the mixture using a sprayer. The fermented mixture was packed using polyethylene stretch film with a compaction of 800 cm^3^ and fermented outdoors for 60 days at 16–30°C using a silage packer (Takakita MW1010H, Japan). Table 1 presents the nutrient compositions of both feeds. Cows were fed *ad libitum* twice daily at 12 h intervals (6:30 and 18:30) with free access to water in individual stall barns. Dairy cows were milked twice daily at 6:00 and 18:00.

**Table 1:** Feed compositions and nutrient levels of two treatment diets.

Item	TMR^a^	FTMR
Nutrient levels^b^		
Dry Matter (DM), % of DM	48.0	46.2
Crude protein (CP), % of DM	17.5	19.1
Neutral detergent fiber (NDF), % of DM	38.3	35.7
Acid detergent fiber (ADF), % of DM	21.5	20.5
Non-fiber carbohydrate (NFC)^c^, % of DM	35.3	34.8
starch, % of DM	21.7	22.4
NE_L_^d^, Mcal/kg of DM	1.61	1.64
Fermentation profile		
pH	6.01	4.69
Lactic acid, % of DM	0.63	8.48
Acetic acid, % of DM	0.64	2.23
NH_3_-N, % of Total N	2.65	5.28

a TMR, total mixed ration; FTMR, fermented total mixed ration.

b Premix contained (DM basis) 14.27% Ca, 5.42% P, 4.96% Mg, 0.05% K, 10.67% Na, 2.98% Cl, 0.37% S, 11 mg/kg Co, 577 mg/kg Cu, 4,858 mg/kg Fe, 51 mg/kg I, 1,806 mg/kg Mn, 13 mg/kg Se, 1,694 mg/kg Zn, 115,240 IU/kg vitamin A, 46,100 IU/kg vitamin D, and 576 IU/kg vitamin E.

c NFC = 100 − NDF − CP − ether extract − ash.

d Calculated based on the Ministry of Agriculture of China (MOA, 2004).

### 2.2 Analysis of milk production and its components

At weeks 7 and 13 of the experiment, milk yield was recorded, and milk samples were collected over 6 consecutive days. Each day, at the Henan Dairy Herd Improvement Testing Center in Zhengzhou, China, a 50 mL aliquot of milk, proportional to the actual daily yield (morning and afternoon), was mixed using an automated near-infrared milk analyzer (MilkoScan, Foss Electric, Hillerød, Denmark). Potassium dichromate was added to preserve the milk samples, which were then stored at 4°C until analysis. Lactose, protein, fat, milk urea nitrogen (MUN), total solids (TS), and somatic cell count (SCC) were determined using infrared analysis methods described by Laporte and Paquin (25).

### 2.3 Fecal sample collection

During weeks 7 and 13 of the experiment, about 500 g of spot fecal samples were gathered from the cows’ rectums using sterile gloves. These samples were composited for each cow at both 6:00 and 18:00 over three consecutive days. To measure the pH, 5 g of fecal material was mixed with 250 mL of distilled water, and the pH was promptly measured using a portable pH meter (PHB-5, ShanghaiLeici, Shanghai, China).

One set of samples was used for total-tract apparent digestibility. Fecal samples were dried at 60°C in a forced-air oven and then ground using a 1 mm screen in a microplant grinding machine (FZ102, Taisite Instrument Co., Ltd., Tianjin, China). Subsequently, they were analyzed for dry matter (DM), crude protein (CP), ash, starch, neutral detergent fiber (NDF), acid detergent fiber (ADF), and indigestible NDF (iNDF). The other set of samples was used for fecal microorganism analysis. The samples were immediately frozen using liquid nitrogen. Equal amounts of frozen samples from each cow and time point were combined to ensure uniformity, using a sterile tap homogenizer (Shanghai Hannuo Ltd., Shanghai, China). These mixed samples were then stored at −80°C to minimize microbial activity, preparing them for subsequent DNA extraction.

### 2.4 Fecal sample analysis

Feed samples were analyzed for ash, dry matter (DM), crude protein (CP), and starch following procedures 942.05, 934.01, 976.05, and 982.30, respectively, as outlined by the Association of Official Analytical Chemists (AOAC, 1990). Neutral detergent fiber (NDF) and acid detergent fiber (ADF) concentrations were determined consecutively using an Ankom A200 fiber analyzer (Ankom Technology, Macedon, NY). Indigestible NDF (iNDF) served as an indirect marker, and total tract apparent nutrient digestibility was calculated accordingly. The iNDF marker was identified through *in vitro* analysis described by Goeser and Combs (26). For fecal samples, raw material was diluted 1:4 with distilled water to measure fecal pH using an Accumet AB150 pH meter (Fisher, Canada).

Fecal volatile fatty acid (VFA) content was determined using high-performance liquid chromatography (HPLC, Waters 600, Milford, Massachusetts, United States). Each sample preparation involved diluting 1 g of fecal material with 1 mL of water. Following this, 300 μL of an internal standard (4-methylvaleric acid, Sigma-Aldrich, St. Louis, MO) and 200 μL of 25% phosphoric acid were added, thoroughly mixed, and then centrifuged at 12,000 × g for 15 min at 4°C. The resulting supernatant was transferred to a new tube for analysis using HPLC, following the method detailed by Wang et al. (27).

### 2.5 Body condition score

During weeks 7 and 13 of the experiment, veterinarians assessed the body condition score (BCS) of the experimental cows shortly after the morning milking session. The assessment used a 5-point scale (1–5 points, with increments of 0.25) as outlined by Vasseur et al. (28).

### 2.6 Amplification of 16S rRNA and Illumina MiSeq sequencing

Genomic DNA extraction from fecal samples utilized the TIANamp Bacteria DNA Kit (TIANGEN, Peking, China) following the manufacturer’s protocol. Verification of DNA concentration and integrity was conducted using both agarose gel electrophoresis and a NanoDrop 2000 spectrophotometer (Thermo Fisher Scientific, Massachusetts, United States). The DNA was subsequently amplified in triplicate employing the Q5 High-Fidelity DNA Polymerase System (New England Biolabs (Beijing) LTD, Beijing, China). The V3–V4 region of the 16S rRNA gene for bacterial analysis was amplified using primers 338F (5′-ACTCCTRCGGGAGGCAGCAG-3′) and 806R (5′-GGACTACCVGGGTATCTAAT-3′) (23), while the 16S rRNA gene region V3–V4 for archaeal analysis was amplified using primers 524F (5′-TGYCAGCCGCCGCGGTAA-3′) and 958R (5′-YCCGGCGTTGAVTCCAATT-3′). PCR amplification consisted of an initial denaturation at 95°C for 3 min, followed by 25 cycles of denaturation at 95°C, annealing at 60°C for bacteria/55°C for archaea, and elongation at 72°C, concluding with a final extension step at 72°C for 10 min. PCR products were analyzed by 2% agarose gel electrophoresis. Purification utilized the AxyPrep DNA Gel Extraction Kit (Axygen Bioscience, Union City, CA, United States), followed by quantification using the Quant-iT PicoGreen dsDNA Assay Kit (Thermo Fisher Scientific, Massachusetts, United States). Sequencing libraries were validated using an Agilent Bioanalyzer (Agilent Technologies, Palo Alto, CA, United States), and their quantification was confirmed by an Agilent Bioanalyzer (Agilent Technologies, Palo Alto, CA, United States) and Promega QuantiFluor^™^-ST (Promega, Madison, WI, United States). Sequencing was performed by Frasergen Bioinformatics Technology Co., Ltd. (Wuhan, China) on an Illumina MiSeq platform (Illumina, Inc., San Diego, CA, United States), and the original RNA-seq data have been deposited into the NCBI Sequence Read Archive (SRA), accession number: PRJNA1119071.

### 2.7 Statistical and bioinformatics analyses

Processing of sequences from the MiSeq platform was performed using QIIME (version 1.8.0) (29). Reads meeting criteria of average quality score ≥ 25 and length between 220–250 nt were retained. Overlapping sequences (>10 bp overlap) were assembled using FLASH v1.2.7. To get high-quality clean tags, raw reads underwent specific filtering conditions via QIIME (v1.8.0) quality control: sequences ≤160 bp or with ≥8 bp homopolymers were excluded. Operational taxonomic units (OTUs) were clustered at 97% identity using UCLUST (30), and chimeric sequences were removed with USEARCH (v5.2.236, http://www.drive5.com/usearch/). The most prevalent sequence within each OTU (bacteria and archaea) was defined as the “representative sequence” and aligned against the SILVA bacterial database (version 119) (31), NCBI-nt protozoa database (32), Unite fungi ITS database (version 7.0) (33), and SILVA archaea database (31) using PyNAST (29) with standard parameters. Alpha diversity indices (ACE, Chao1, Shannon, Simpson) were computed by rarefied samples in QIIME to assess diversity and abundance of bacterial and archaeal communities. Principal component analysis (PCA) was utilized for beta diversity evaluation. Linear discriminant analysis effect size (LefSe) analysis on the Galaxy online platform (34) identified discriminative functional biomarkers, employing a size-effect threshold of 2.0 on the logarithmic LDA score to determine major abundant modules in the TMR and FTMR groups.

Independent-sample *t*-tests (for normally distributed data) or Mann–Whitney U tests (for nonnormally distributed data) were used to determine substantial differences in the relative abundance of the top 10 phyla and genera and in the alpha diversity indices between the two groups. *p* < 0.05 and *p* < 0.01 indicated statistically significant and extremely significant differences, respectively.

## 3 Results

### 3.1 Effect of TMR and FTMR on the lactation performance of dairy cows

Table 2 presents milk production and component findings in addition to feed intake. Dairy cows consuming the FTMR diet showed reduced DMI, increased milk fat concentration, higher ECM, and improved feed efficiency compared to those on the TMR diet. Significant differences in feed efficiency were observed between the TMR and FTMR groups.

**Table 2:** Effects of feeding two treatment diets based on TMR and FTMR diets on lactation performance in dairy cows.

Item^b^	Treatment^a^		SEM	*p*-value
	TMR	FTMR		
The 7th week				
DMI, kg/d	20.7	17.3	0.73	0.01
Milk Yield, kg/d	28.1	29.5	0.94	0.35
ECM^c^ Yield, kg/d	30.2	31.7	0.30	0.01
Fat%	3.72	3.98	0.06	0.01
Protein%	3.30	3.33	0.06	0.67
Lactose%	4.77	4.82	0.05	0.82
Total solids%	12.5	11.8	0.27	0.20
Feed efficiency^d^	1.37	1.71	0.08	0.01
SCC, ×10 cells/mL	47.5	81.3	26.4	0.59
Body scores	2.96	3.00	0.06	0.88
The 13th week				
DMI, kg/d	18.9	17.1	0.46	0.03
Milk Yield, kg/d	29.0	29.4	0.82	0.80
ECM Yield, kg/d	30.4	31.7	0.26	0.01
Fat%	3.81	3.96	0.03	0.01
Protein%	3.35	3.34	0.06	0.77
Lactose%	4.87	4.80	0.05	0.57
Total solids%	12.9	12.3	0.37	0.48
Feed efficiency^d^	1.55	1.72	0.04	0.03
SCC, ×10^3^ cells/mL	70.1	51.1	20.3	0.99
Body scores	2.92	3.04	0.07	0.41

a TMR, total mixed ration; FTMR, fermented total mixed ration; DMI, dry matter intake; SCC, somatic cell count; ECM, energy corrected milk.

b ECM (Kg) = 0.3246 × milk yield (kg) + 13.86 × fat yield (kg) + 7.04 × protein yield (kg).

c Feed efficiency = ECM/DMI.

### 3.2 Effect of TMR and FTMR on the apparent total tract digestibility of dairy cow feces

Table 3 shows the apparent total tract nutrient digestibility of the two treatment diets, which was greater for the FTMR diet than for the TMR diet for DM, OM, CP, NDF and ADF.

**Table 3:** Effects of feeding two treatment diets based on TMR and FTMR diets on total-tract apparent digestibility in dairy cows.

Item	Treatment^a^		SEM	*p*-value
	TMR	FTMR		
The 7th week, %				
DM	68.9	75.3	0.89	0.00
OM	70.1	75.7	0.94	0.01
CP	76.4	80.2	0.99	0.03
NDF	41.2	51.7	1.19	0.00
ADF	36.1	49.3	1.72	0.00
The 13th week, %				
DM	71.5	75.2	0.95	0.01
OM	71.7	75.2	1.09	0.04
CP	78.4	82.2	1.25	0.04
NDF	45.2	49.6	0.91	0.01
ADF	42.8	46.6	0.95	0.05

a TMR, total mixed ration; FTMR, fermented total mixed ration; OM, organic matter; DM, dry matter; CP, crude protein; NDF, neutral detergent fiber; ADF, acid detergent fiber.

### 3.3 Effect of TMR and FTMR on the fecal VFA pattern and pH of dairy cows

The fecal pH, acetate, and propionate were lower (*p* < 0.05) in the FTMR-treated samples than in the TMR-treated samples (Table 4). The content of butyrate in the feces of cows fed the FTMR was greater than that in the feces of cows fed the TMR. No significant effects were observed for fecal total VFA concentration, isobutyrate, valerate, isovalerate, and acetate/propionate ratio.

**Table 4:** Fecal VFA pattern and pH of cows fed two different diets of TMR and FTMR.

Items	Treatment^a^		SEM	*p*-value
	TMR	FTMR		
Fecal pH	6.7	6.36	0.07	0.03
Total VFA (μmol/g)	26.04	26.29	0.87	0.12
Acetate (μmol/g)	19.33	18.17	0.31	0.03
Propionate (μmol/g)	4.00	3.45	0.15	0.03
Butyrate (μmol/g)	2.18	3.88	0.21	0.001
Isobutyrate (μmol/g)	0.180	0.177	0.01	0.35
Valerate (μmol/g)	0.190	0.187	0.02	0.42
Isovalerate (μmol/g)	0.156	0.177	0.05	0.36
Acetate/Propionate	4.833	5.262	0.14	0.06

a TMR, total mixed ration; FTMR, fermented total mixed ration.

### 3.4 Analysis of 16S rRNA sequencing of bacteria and archaea in the feces of dairy cows fed the TMR and FTMR

**Figure 1:**
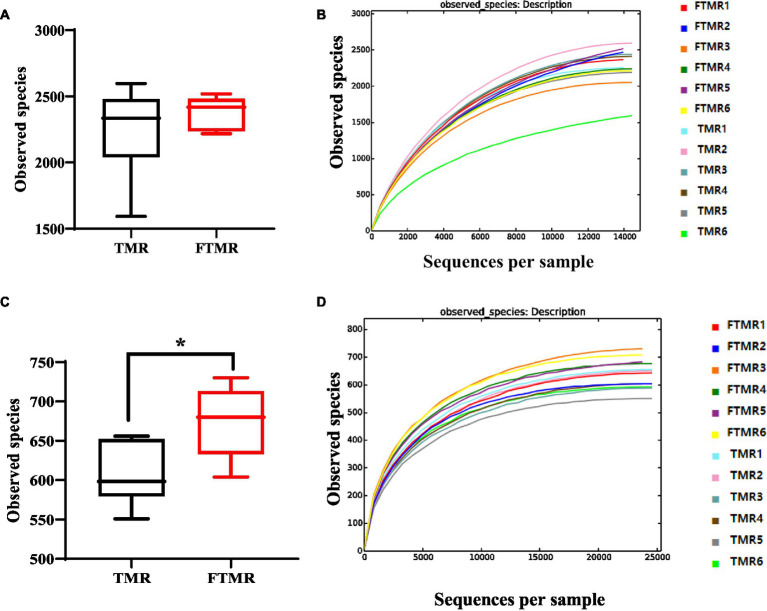
Boxplot and rarefaction curves of operational taxonomic units (OTUs). Bacterial **(A)** and archaea **(C)** boxplots represent the number of observed OTUs. The *x*-axis shows the observed species (OTUs), and the *y*-axis shows the relationship between TMR and FTMR. The OTU similarity threshold of 97% was considered. Boxes represent the interquartile range (IQR) between the first and third quartiles (25th and 75th percentiles, respectively), and the horizontal line inside the box defines the median. Whiskers represent the lowest and highest values within 1.5 times the IQR from the first and third quartiles. ^*^p < 0.05 (Student’s *t*-test). Bacterial **(B)** and archaea **(D)** rarefaction curves of OTUs. The *x*-axis shows the number of valid sequences per sample, and the *y*-axis shows the observed species (OTUs). Each curve in the graph represents a different sample and is shown in a different color. As the sequencing depth increased, the number of OTUs also increased. Eventually, the curves began to plateau, indicating that as the number of extracted sequences increased, the number of OTUs detected decreased.

Figure 1A displays bacterial species (OTUs) found in TMR and FTMR, and Figure 1C illustrates archaeal species (OTUs). Cows fed FTMR exhibited significantly more observed species than those fed TMR (*p* < 0.05).

The sparsity curves of the OTUs identified in this study indicated that as sequencing depth increased, more species were detected. With higher numbers of sequences analyzed, the edges of the sparsity curves flattened, suggesting thorough coverage of the sequencing data (Figures 1B,D). Good’s coverage, which assesses how well samples are represented by sequencing, approached nearly 99%, indicating comprehensive detection of bacterial types in the samples.

### 3.5 Differences in bacterial and archaeal diversity between the feces of dairy cows fed the TMR and FTMR

The alpha diversity indices commonly employed included abundance indices (Chao1 and ACE) and diversity indices (Simpson and Shannon). Analysis of fecal bacteria between the two groups indicated no statistically significant differences in the Chao1, ACE, Shannon, or Simpson indices, as indicated in Table 5 (*p* > 0.05). Moreover, FTMR significantly increased the fecal archaeal abundance indices (Chao and ACE indices) and diversity indices (Shannon index) (*p* < 0.05).

**Table 5:** Changes in fecal microbial richness and diversity between TMR and FTMR groups.

Item	Treatment^a^		SEM	*p*-value
	TMR	FTMR		
Bacteria				
Chao 1	2316.88	2447.39	121.98	0.47
ACE	2335.68	2531.16	151.06	0.38
Shannon	9.59	9.49	0.20	0.74
Simpson	0.993	0.991	0.001	0.33
Archaea				
Chao 1	607.51	676.74	17.74	0.02
ACE	609.37	680.44	18.67	0.02
Shannon	5.47	5.96	0.05	0.01
Simpson	0.902	0.928	0.007	0.25

a TMR, total mixed ration; FTMR, fermented total mixed ration.

**Figure 2:**
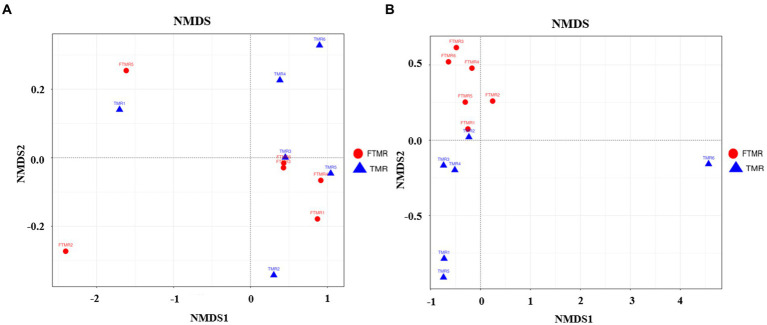
NMDS of fecal microbial communities. Weighted NMDS by fecal bacteria **(A)** and archaea **(B)**. TMR, total mixed ration; FTMR, fermented total mixed ration.

Beta diversity evaluations were used to analyze the similarities in community structure between the two groups. The NMDS analysis highlighted significant differences in fecal bacterial communities, clearly distinguishing between the TMR and FTMR groups at the OTU level. Similarly, the NMDS results showed substantial variability in fecal archaeal communities, with a considerable separation observed between the two groups, indicating distinct compositions of archaeal communities in the TMR and FTMR groups (See Figure 2).

### 3.6 Relative abundance and core microbiota of bacteria and archaea in the feces of dairy cows fed the TMR and FTMR

**Figure 3:**
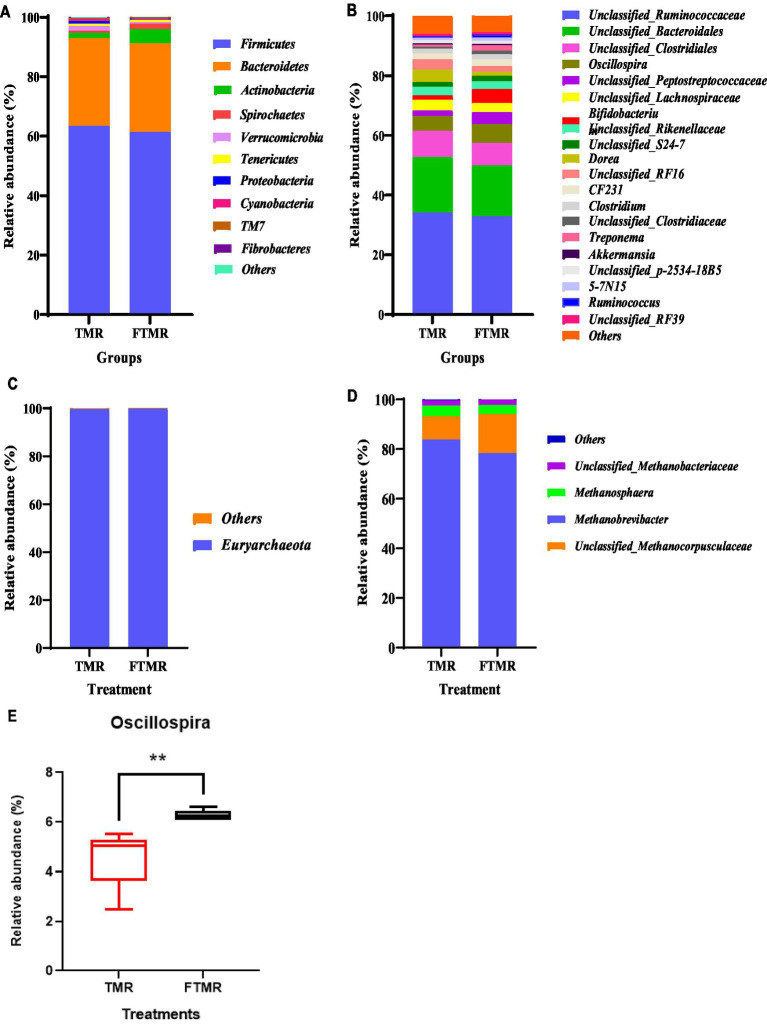
Relative fecal microbial abundances at phylum and genus levels. The abundances of phyla of bacteria **(A)** and archaea **(C)**. The abundances of genera of bacteria **(B)** and archaea **(D)**. *Oscillospira* genus **(E)** between both groups. Small box plots show the 25th, 50th and 75th percentiles, and whiskers show the extreme values of the data. TMR, total mixed ration; FTMR, fermented mixed ration.

Figure 3 displays the distributions of the top 10 and 20 phyla and genera of fecal bacteria in the TMR and FTMR groups. Bacterial phyla with a relative abundance exceeding 1% were considered predominant. At the phylum level (Figure 3A), *Firmicutes*, *Bacteroidetes*, and Actinobacteria were prevalent in both groups. Among genera with a relative abundance exceeding 1% (Figure 3B), dominant bacteria included *Unclassified_Ruminococcaceae*, *Unclassified_Bacteroidales*, *Unclassified_Clostridiales*, *Oscillospira*, *Unclassified_Peptostreptococcaceae*, *Unclassified_Lachnospiraceae*, *Bifidobacterium*, *Unclassified_Rikenellaceae*, *Unclassified_S24-7*, *Dorea*, *Unclassified_RF16*, *CF231*, *Clostridium*, and *Unclassified_Clostridiaceae*. *Oscillospira* exhibited a notable difference between the TMR and FTMR groups (*p* = 0.01) (Figure 3E).

**Figure 4:**
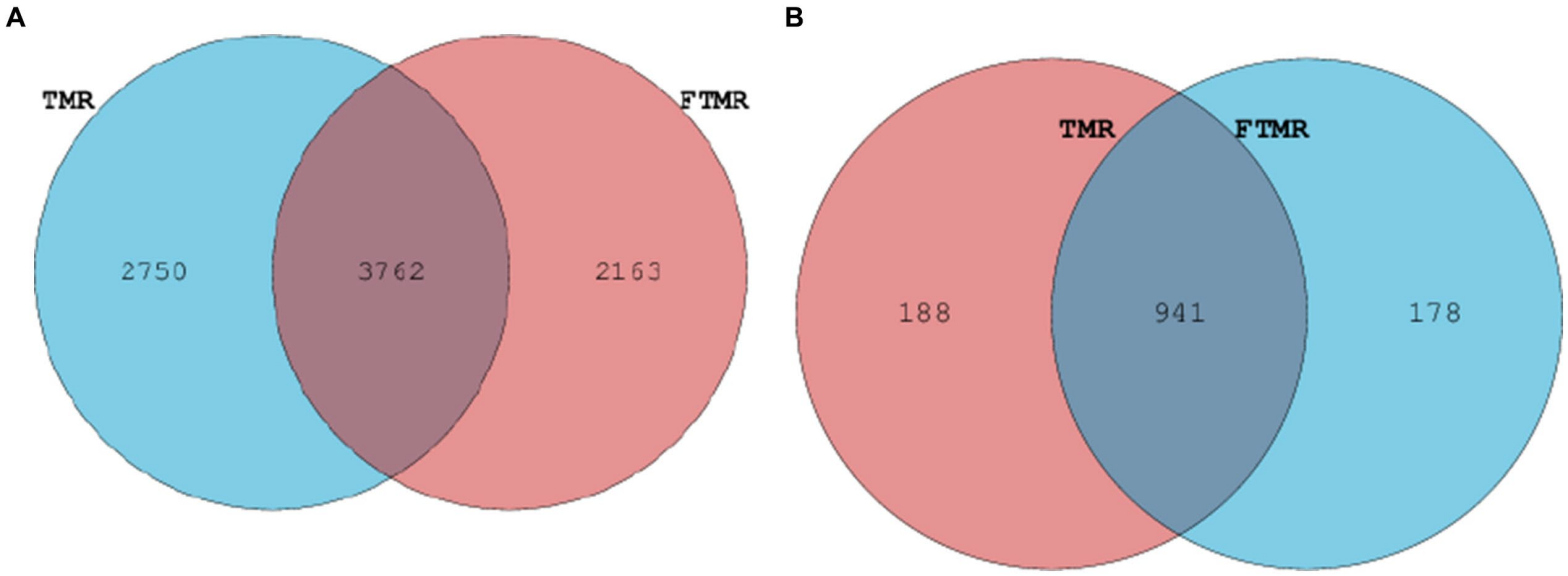
Venn diagram illustrating overlapping microbial OTUs at 3% dissimilarity level between both groups. Venn diagram of bacteria **(A)** and archaea **(B)** OTUs. TMR, total mixed ration; FTMR, fermented total mixed ration.

Figure 3 presents the relative abundances of fecal archaea in the TMR and FTMR groups at both the phylum and genus levels. Archaeal phyla with a relative abundance exceeding 1% were classified as dominant. At the phylum level (Figure 3C), *Euryarchaeota* were the most prevalent archaea in both groups. Among archaeal genera with relative abundances exceeding 1% (Figure 3D), dominant archaea included *Methanobrevibacter*, *Unclassified_Methanocorpusculaceae*, *Methanosphaera*, and *Unclassified_Methanobacteriaceae*. Taxa shared between the TMR and FTMR groups were considered part of the core microbial community. The number of shared OTUs for bacteria (Figure 4A) was 3,762, and for archaea (Figure 4B), it was 947.

### 3.7 LDA effect size analysis between the TMR and FTMR groups

**Figure 5:**
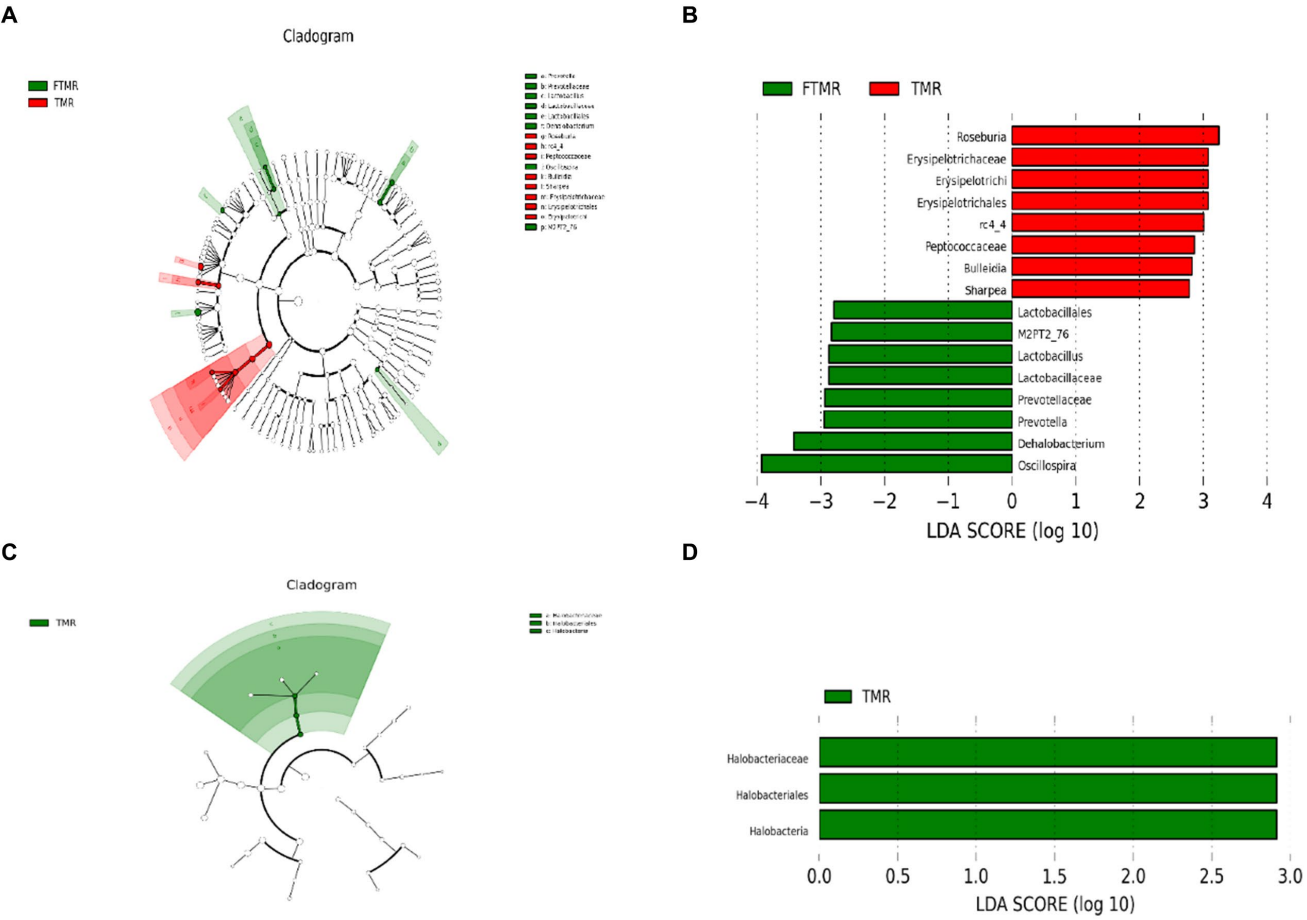
Effect size (LefSe) analysis for LDA. Cladogram diagram showing the microbial species with remarkable variation between TMR and FTMR groups. Cladogram diagram of bacteria **(A)** and archaea **(C)**; species exhibiting remarkable differences with linear discriminant analysis (LDA) > 2.0, histograms of bacteria **(B)** and archaea **(D)**. The length of the histogram denotes the LDA score, which contrasts the degree of impact by species exhibiting noteworthy differences between groups. TMR, total mixed diet; FTMR, fermented total mixed diet.

The enrichment module rankings were determined using LEfSe analysis. The cladogram (Figure 5A) visually confirmed differences in 16 bacterial taxa between the TMR and FTMR groups. Figure 5B, the LEfSe plot, displays the varying LDA scores of bacterial taxa between these groups. Significant biomarkers for bacteria included *Roseburia*, *rc4-4*, *Bulleidia*, and *Sharpea* at the genus level. Similarly, notable biomarkers for archaea between the groups were *Lactobacillus*, *Prevotella*, *Dehalobacterium*, and *Oscillospira*.

Figure 5C displays variations among the three archaeal taxa between the TMR and FTMR groups in a cladogram. In Figure 5D, the LEfSe analysis plot illustrates significant differences in LDA scores of archaeal taxa between these groups. Significant biomarkers distinguishing between the groups were evident at the class and order levels, specifically *Halobacteria* and *Halobacteriales*, respectively. Additionally, at the family level, *Halobacteriaceae* showed significant differences between the TMR and FTMR groups.

## 4 Discussion

### 4.1 Lactation performance, total tract apparent digestibility, fecal pH, and VFA of dairy cows

DMI is a crucial factor influencing dairy cow production performance (35). In this study, a decrease in DMI was observed in the FTMR group, while milk yield remained unaffected. This indicates that the FTMR diet demonstrates superior feed efficiency. The concentration of acetic acid in relation to total acids was negatively associated with silage intake (36). Previous research has shown that acetic acid concentrations exceeding 1.7% DM significantly reduce ruminant intake (37). Additionally, ammonia-N concentration in grass silage has been inversely correlated with dry matter intake in lactating dairy cows (38). As a result, the FTMR-fed group exhibited higher levels of organic acids and ammonia nitrogen, along with reduced dry matter intake compared to the TMR-fed group. However, FTMR had no impact on DMI in Ujumqin sheep, likely due to species differences and diet fermentation quality (39). Despite decreased DMI in the FTMR diet, milk yield was unaffected due to improved feed efficiency and increased ECM compared to the TMR diet. Previous research indicates that exogenous fibrolytic enzymes enhance fiber digestibility, thereby increasing available energy intake and improving feed efficiency (40). Compared to the TMR diet, FTMR demonstrated higher nutrient digestibility and feed efficiency, notably increasing DM digestibility (41). Cao et al. observed increased DM digestibility with fermented TMR in sheep compared to regular TMR consumption (39). Additionally, CP and NDF digestibility were enhanced in the FTMR diet, consistent with recent studies demonstrating improved CP and NDF digestibility in sheep fed FTMR, attributed to increased digestible CP and NDF levels (42). Enzyme levels have been reported to significantly impact total tract DM and NDF digestibility in a cubic manner (43), thereby equivalently elevating OM digestibility in cows fed the FTMR diet. Acetate is recognized as a primary precursor for milk fat synthesis in the mammary gland during lactation (44). Previous studies have shown that sheep fed FTMR exhibited increased acetate concentrations compared to those fed fresh TMR (39), contributing to increased milk fat production due to enhanced fiber digestibility from added fibrolytic enzymes (43).

Fermentation altered the carbohydrate composition in the FTMR, affecting hindgut fermentation patterns. Unlike the TMR group, the FTMR group showed a lower fecal pH. Adding starch (soluble carbohydrate) to dairy cow diets can significantly reduce fecal pH, potentially increasing nutrient bypassing in the rumen and accelerating passage rates (45). Hindgut microbes in dairy cows degrade 5 to 10% of carbohydrates, including starch, small particles escaping ruminal fermentation, and undigested rumen components (46, 47). After ensiling, the TMR exhibited reduced nonfibrous carbohydrate content, suggesting that FTMR increases concentrations of nonfibrous and soluble carbohydrates, speeds up feed passage rates, and boosts fermentable substances in the hindgut. Studies have reported that diets high in grains (rich in non-fiber carbohydrates) significantly increase fecal butyric acid levels and lower pH in cows (48), which is consistent with the current study’s findings. The FTMR group showed markedly higher fecal butyrate concentrations, corresponding with an increased relative abundance of key butyrate-producing bacteria like *Ruminococcaceae*, *Oscillospira*, and *Bacteroidales*. Acetate and propionate are crucial energy sources for ruminants. Research suggests that dietary fermentation promotes propionate fermentation and absorption in the rumen (49, 50), potentially influencing microbial populations producing these acids in both the rumen and hindgut.

### 4.2 Fecal bacterial community composition of dairy cows

The relative abundance histogram of phyla indicated that *Firmicutes*, along with *Bacteroidetes*, were the predominant fecal bacteria, consistent with prior studies (51–53). Previous research has linked the *Firmicutes* to *Bacteroidetes* ratio with energy extraction in humans and mice (54, 55). Our findings showed no change in the *Firmicutes* to *Bacteroidetes* ratio, suggesting that this ratio remains largely unaffected by ruminant diets (56). This could be attributed to the primary energy production occurring in the foregut rather than the hindgut (20).

In the feces, the dominant genera included *Unclassified_Bacteroidales*, *Unclassified_Ruminococcaceae*, *Unclassified_Clostridiales*, and *Oscillospira*. Previous studies have highlighted that *Unclassified_Ruminococcaceae* and *Unclassified_Bacteroidales* are integral parts of the rumen’s “core bacterial microbiome” (57). These genera play crucial roles in breaking down plant fibers within the gastrointestinal tract (57).

In this study, the abundance of *Unclassified_Clostridiales*, considered a core component of fecal bacteria, did not exhibit significant differences between the TMR and FTMR groups, which is consistent with prior research (58). This indicates that *Unclassified_Clostridiales* may contribute significantly to the fecal microbial ecosystem regardless of dietary differences. Further investigation involving the isolation and identification of representative strains of *Unclassified_Clostridiales* could provide deeper insights into their role in hindgut function (59).

Our findings revealed a significantly higher abundance of *Oscillospira* in the FTMR group compared to other groups at the genus level. The content of *Oscillospira* in the rumen of cattle fed a high-starch (soluble carbohydrates) diet increased (60). Zhao et al. suggested that fermentation increases the crystalline structure of starch grains, promoting the production of resistant starch (61). Therefore, the elevated levels of *Oscillospira* in the FTMR group may be attributed to the higher content of resistant starch in the hindgut of cows fed FTMR. Mackie et al. noted that *Oscillospira* is prevalent in the rumen of dairy cows and influenced by dietary factors (62), indicating its responsiveness to diet in the gastrointestinal tract. *Oscillospira* is known as a potential butyrate producer, contributing to intestinal health (63), suggesting that FTMR diets may enhance gastrointestinal health through increased *Oscillospira* abundance. Although *Oscillospira* has not been cultured in pure form (9), its potential as a probiotic for regulating the gastrointestinal tract of dairy cows merits consideration.

At the genus level, *Roseburia*, *rc4-4*, *Bulleidia*, and *Sharpea* were significantly more abundant in the TMR group compared to the FTMR group, consistent with findings by Kim et al. (58). This indicates that less common bacterial taxa are more responsive to dietary changes than those more commonly found. *Roseburia* species are known for their ability to break down dietary polysaccharides, producing butyrate (64), which correlates with our observation of higher butyric acid levels in the FTMR group than in the TMR group. The genus *rc4-4* is associated with diets rich in fiber (65), suggesting that the TMR diet may have a higher fiber content in the hindgut compared to the FTMR diet. *Sharpea* plays a critical role in lactic acid production (66, 67), which increases during rumen acidosis in cows (68) and varies with diet composition in sheep (69). While lactic acid is not a primary product of normal rumen fermentation, diets high in carbohydrates and soluble sugars can lead to its accumulation (70). Hence, the higher abundance of *Sharpea* in the TMR group may be due to greater carbohydrate flow and soluble sugar levels from the rumen into the hindgut.

We identified four genera—*Lactobacillus*, *Prevotella*, *Dehalobacterium*, and *Oscillospira*—as distinguishing biomarkers between the FTMR and TMR groups. *Lactobacillus* is recognized for its probiotic role in ruminant gastrointestinal health and has been associated with reducing diarrhea rates in calves (71–73). Our findings showed that *Lactobacillus* constituted less than 1% of the fecal microbiota and was not considered a core community, consistent with Tang et al. (74). Its abundance varies with diet, transitioning from lactose dominance in younger animals to diets richer in complex carbohydrates as they mature (75–77). While *Lactobacillus* thrives in silage, its survival through the gastrointestinal tract, particularly in the rumen, is limited (50, 74). Nonetheless, our results indicated significantly higher levels of *Lactobacillus* in the feces of the FTMR group compared to the TMR group, suggesting silage could serve as a vehicle for probiotic delivery (50). *Prevotella* was abundant in both TMR and FTMR groups, consistent with its widespread presence in cow feces (56, 78, 79) and dominance in the rumen (57, 71, 80). Its prevalence in feces (81) varies with diet, such as higher levels in corn-based diets compared to those with wet distillers grains (82), indicating its diet-related presence in cattle feces (58, 83, 84). *Prevotella* species are known for their enzymatic capabilities in degrading proteins, starch, and hemicellulose to produce succinate and acetate (85–87). *Dehalobacterium* showed a higher abundance in FTMR feces compared to TMR, suggesting diet influences its presence (88). It has been linked to glucose-rich diets, although further research is needed on its role in the hindgut of dairy cows (89).

### 4.3 Fecal archaeal community composition of dairy cows

Our results showed that the archaeal Chao1 index in the FTMR group was markedly greater than that in the TMR group, confirming that the archaeal diversity in the FTMR group was markedly greater than that in the other groups. The predominant archaeal phylum identified in the feces was *Euryarchaeota*. Prior investigations have indicated that *Euryarchaeota* is the most prevalent archaeal bacterial phylum in the rumen (22, 85, 90), underscoring its critical function in the gastrointestinal system. It was further confirmed that *Methanobrevibacter* is the most prevalent archaeal bacterial genus in feces, aligning with previous studies (22, 91), which established their capability to generate CH_4_ from hydrogen and formic acid (91, 92). Nevertheless, no significant differences in *Methanobrevibacter* abundance were observed between the FTMR and TMR groups, implying that FTMR may not diminish methane production in the hindgut. Zhang et al. (93) noted that high-concentrate rations significantly decreased the abundances of *Fibrobacter* and *Methanobrevibacter*, demonstrating that an increase in these bacteria’s concentration in the diet could inhibit methane synthesis (94, 95). Conversely, this study did not alter the concentration-to-coarse ratio between TMR and FTMR. Moreover, a symbiotic interaction exists between bacteria and methanogens in the animal gut, and cellulolytic bacteria in the rumen of ruminants show a positive correlation with the number of methanogens (96). Notably, no significant alterations in the relative abundances of *Fibrobacteres* and *Fibrobacter* were detected in this study between the TMR and FTMR groups. *Halobacteria*, *Halobacteriales*, and *Halobacteriaceae* served as biomarkers to differentiate TMRs from FTMR, with their relative abundances being considerably higher in the TMR group than in the FTMR group, potentially due to variations in hindgut cell chloride content between the two groups (97).

## 5 Conclusion

In conclusion, FTMR was shown to increase milk fat content, improve nutrient digestibility, elevate the proportion of butyrate in fecal matter, and decrease fecal pH. These findings indicate that FTMR can increase hindgut fermentation. Additionally, FTMR significantly enhanced the diversity of fecal archaeal communities and increased the relative abundance of the genus *Oscillospira*, suggesting improved hindgut health compared to TMR. While *Methanobrevibacter* was found to be the most common archaeal genus in fecal samples, no significant disparities were observed between the TMR and FTMR groups, suggesting that FTMR may not have a significant impact on methane production in the hindgut. This study provides a theoretical basis for investigating the impact of FTMR on bacterial and archaeal communities in the hindgut, indicating that FTMR feeding can beneficially affect hindgut health in dairy cows.

## Data availability statement

The data presented in the study are deposited in the NCBI Sequence Read Archive (SRA), accession number: PRJNA1119071.

## Ethics statement

The animal study was approved by Animal Care Advisory Committee, Henan Agricultural University. The study was conducted in accordance with the local legislation and institutional requirements.

## Author contributions

LW: Writing – original draft, Writing – review & editing, Conceptualization. SJ: Investigation, Software, Writing – review & editing. PW: Data curation, Writing – original draft. XL: Methodology, Writing – review & editing. CL: Formal analysis, Supervision, Writing – review & editing. SS: Formal analysis, Writing – review & editing. GZ: Project administration, Writing – review & editing. JC: Resources, Writing – review & editing. QY: Funding acquisition, Writing – review & editing. HZ: Resources, Visualization, Writing – original draft. QZ: Supervision, Writing – review & editing.
